# Angiography-derived fractional flow and clinical outcomes in symptomatic intracranial atherosclerotic stenosis

**DOI:** 10.3389/fneur.2026.1944177

**Published:** 2026-10-15

**Authors:** Wanning Zheng, Rong Zou, Ming Wang, Jiahao Tang, Ruilin Chen, Chaobo Zheng, Jie Gong, Yuhai Gao, Ning Wang, Yuning Lu, Jiong Wu, Jens Fiehler, Adnan H. Siddiqui, Jianping Xiang, Shu Wan

**Affiliations:** 1 Brain Center, Zhejiang Hospital, Hangzhou, China; 2 Zhejiang Province Engineering Research Center for Precision Medicine in Cerebrovascular Diseases, Hangzhou, China; 3 ArteryFlow Technology Co., Ltd., Hangzhou, China; 4 The Second School of Clinical Medicine, Zhejiang Chinese Medical University, Hangzhou, China; 5 Department of Diagnostic and Interventional Neuroradiology, University Medical Center Hamburg-Eppendorf, Hamburg, Germany; 6 Department of Neurosurgery, University of Buffalo, Buffalo, NY, United States

**Keywords:** angiography, cerebrovascular circulation, cerebrovascular disorders, digital subtraction, endovascular procedures, intracranial arteriosclerosis

## Abstract

**Background:**

To explore the association between fractional flow (FF) and clinical outcomes in patients with symptomatic intracranial atherosclerotic stenosis (ICAS), and to evaluate its potential role in patient stratification.

**Methods:**

This retrospective exploratory study included symptomatic ICAS patients hospitalized between March 2019 and May 2024. FF values were derived from digital subtraction angiography using an angiography-based computational method. Patients were stratified according to a pre-specified FF threshold of 0.76 and treatment modality (interventional vs. conservative). Clinical outcomes were assessed using the modified Rankin Scale (mRS) and National Institutes of Health Stroke Scale (NIHSS) at discharge and 12 months.

**Results:**

Among patients with FF ≤ 0.76, no worsening in mRS between discharge and 12 months was observed in 96.3% of the interventional group and 78.6% of the conservative group (*p* = 0.017). In a *post hoc* analysis, improvement of at least one mRS point occurred in 46.3 and 17.9%, respectively; however, discharge mRS scores differed between groups, and the difference in improvement was not statistically significant when the analysis was restricted to patients with discharge mRS ≥ 1. No significant differences in mRS or NIHSS improvement were observed between treatment strategies in patients with FF > 0.76 (*p* > 0.05). In contrast, stratification based on degree of stenosis (DS% ≥ 70%) did not differentiate clinical outcomes between treatment groups. These findings suggest that FF-based stratification may be associated with differences in functional outcomes across treatment strategies.

**Conclusion:**

FF may provide additional information for risk stratification in symptomatic ICAS patients and may help identify clinically relevant subgroups for further evaluation. However, these findings should be considered exploratory and require validation in larger prospective studies.

## Introduction

Stroke remains a leading cause of death and disability worldwide (1), with ischemic stroke accounting for the majority of cases (2). Intracranial atherosclerotic stenosis (ICAS) is a major contributor to ischemic stroke, particularly in Asian populations, where it accounts for up to 30–50% of cases (3). Current treatment strategies for ICAS primarily include aggressive medical therapy and endovascular intervention. Large randomized trials, such as SAMMPRIS and VISSIT, have established medical therapy as the first-line treatment for ICAS. However, despite optimal medical management, patients with severe ICAS still face a substantial risk of recurrent stroke, with an annual recurrence rate of approximately 9–12% (4, 5). More recent studies have suggested that selected patients may benefit from endovascular treatment, highlighting the need for improved patient selection strategies (6, 7).

Traditionally, treatment decisions in ICAS have been largely based on the degree of stenosis (DS%). However, DS% alone may not adequately reflect the hemodynamic significance of a lesion. Previous studies, including CICAS and WASID, have demonstrated that stroke risk is not solely determined by the severity of luminal narrowing (8, 9). In addition, hemodynamic factors such as collateral circulation and cerebral perfusion status have been shown to play a critical role in stroke risk and recurrence (10, 11). These findings suggest that functional assessment may provide additional value beyond anatomical measurements in evaluating ICAS.

In this study, fractional flow (FF) denotes the angiography-derived ratio of pressure distal to proximal to the intracranial stenosis under resting conditions. This differs from coronary fractional flow reserve (FFR), which is assessed under maximal hyperemia (12). Similarly, early studies by Miao et al. and Han et al. demonstrated the feasibility of measuring intracranial FF using pressure wires (13, 14). However, the invasive nature of pressure-wire measurements limits their widespread clinical use. More recently, computational fluid dynamics (CFD)-based approaches have enabled estimation of FF from angiographic images without a pressure wire. Several studies have demonstrated good agreement between CFD-derived FF and invasive measurements, supporting its feasibility and reliability in clinical practice (15–17).

However, despite these advances, whether FF can be used to stratify patients and assist in treatment selection for ICAS remains unclear. In particular, there is currently no widely accepted FF threshold to guide clinical decision-making. In coronary artery disease, FFR thresholds ranging from 0.75 to 0.80 have been widely adopted for clinical decision-making. In prior validation studies of angiography-derived FF, a value of 0.76 showed the highest agreement with guidewire-based measurements. Accordingly, 0.76 was adopted as a reference value for stratification in the present study (18). However, this value has not yet been validated in outcome-based clinical studies.

In this study, we conducted a retrospective exploratory analysis to investigate the association between FF and clinical outcomes in patients with symptomatic ICAS. We further explored whether FF-based stratification may help identify patient subgroups with differential responses to treatment strategies.

## Materials and methods

### Study design and population

This was a retrospective exploratory study including symptomatic ICAS patients hospitalized at Zhejiang Hospital between March 2019 and May 2024. Clinical data were accessed for research purposes between June 2025 and July 2025.

#### Inclusion criteria

1. DSA indicated intracranial artery stenosis (including the C4 segment of the internal carotid artery, the V4 segment of the vertebral artery, or above).
2. A history of ischemic stroke or transient ischemic attack (TIA).
3. Availability of pretreatment DSA images of sufficient quality for retrospective FF calculation using AccuFFicas.

#### Exclusion criteria

1. Non-atherosclerotic intracranial artery stenosis, complete vascular occlusion, or acute thrombectomy.
2. Acute cerebral infarction within two weeks prior to the study.
3. Known allergies to contrast agents or other contraindications.
4. Presence of proximal or distal aneurysms in the stenotic segment.
5. Concomitant severe intracranial or extracranial stenosis with tandem vascular lesions.
6. Severely tortuous cerebral vascular lesions.
7. Pregnancy.
8. Patients unable to provide informed consent.

This study was approved by the Medical Ethics Committee of Zhejiang Hospital, and informed consent was obtained from all patients.

### Patient grouping and variables

Patients were stratified according to pretreatment FF values using a pre-specified threshold of 0.76. Based on FF values and treatment modality, patients were categorized into four groups:

1. FF ≤ 0.76 with interventional treatment.
2. FF ≤ 0.76 with conservative management.
3. FF > 0.76 with interventional treatment.
4. FF > 0.76 with conservative management.

Treatment decisions (interventional vs. conservative) were made based on comprehensive clinical judgment, including patient symptoms, imaging findings, and physician experience, rather than FF values alone. In this study, endovascular treatment comprised intracranial stenting or balloon angioplasty alone; these procedures were pooled as the endovascular treatment group in the primary analyses. In addition, a subgroup analysis was performed in patients with degree of stenosis (DS%) ≥ 70%, a commonly used threshold for severe stenosis in clinical practice. Baseline variables included demographic characteristics (age and sex), vascular risk factors (hypertension, diabetes, hyperlipidemia, coronary artery disease), lifestyle factors (smoking and alcohol consumption), and clinical scores (NIHSS and mRS).

### Treatment and follow-up

All patients were prescribed dual antiplatelet and lipid-lowering therapy. For patients undergoing endovascular treatment, the procedure was performed at least 2 weeks after the qualifying ischemic event. The duration of dual antiplatelet therapy varied according to treatment type and clinical judgment; prolonged dual antiplatelet therapy was not routinely prescribed after conservative management or balloon angioplasty alone. At follow-up, all patients reported adherence to the prescribed medical therapy; adherence was assessed by patient report and was not independently verified. Clinical outcomes were assessed at discharge and at follow-up targeted approximately 12 months after discharge. No worsening was defined as an unchanged or lower follow-up mRS or NIHSS score. In *post hoc* analyses, mRS change was classified as improvement (≥1-point decrease), unchanged, or worsening (≥1-point increase); functional independence was defined as a 12-month mRS score of 0–2.

### Measurement of FF value

Unlike coronary artery disease—where vasodilators such as adenosine are routinely administered to achieve maximal hyperemia—ICAS does not require pharmacological vasodilation for FF assessment. This distinction arises from fundamental differences in vascular anatomy and physiology: (1) the intracranial vasculature exhibits greater geometric complexity, with highly variable branching patterns and lumen diameters compared to coronary arteries; and (2) the cerebral circulation maintains robust autoregulatory mechanisms that stabilize blood flow across a wide range of vascular resistances (19). Consequently, FF measurements in ICAS are performed under resting conditions, capturing the unique hemodynamic profile intrinsic to the cerebrovascular system.

The AccuFFicas software is a novel method for reconstructing three-dimensional (3D) intracranial arteries from two-dimensional (2D) DSA images. This software can calculate patient-specific FF values based on hemodynamic parameters, which has been validated as both feasible and reliable (18) (Figure 1).

**Figure 1:**
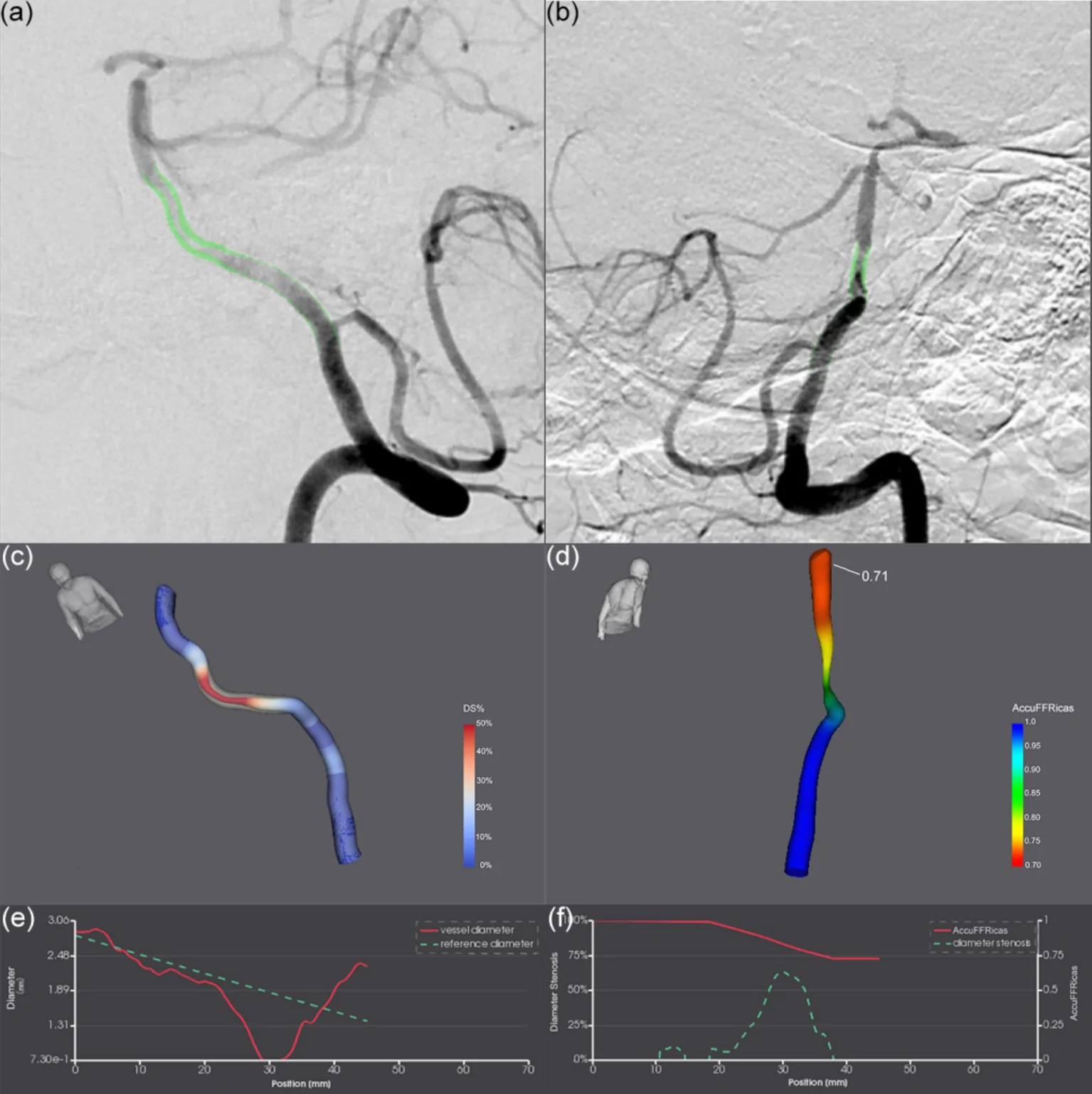
3D model reconstruction based on 3D angiography of intracranial arterial stenosis. **(a,b)** Multidimensional DSA images of intracranial arterial stenosis. **(c)** Segmented 3D reconstructed vessel diameter and stenosis ratio. **(d)** Distribution of pressure ratio (PR) results obtained from computational fluid dynamics analysis. **(e)** Comparison of vessel diameter and reference diameter at different segments. **(f)** Comparison of FF values and direct stenosis severity distribution at different segments.

FF values were calculated using the dedicated AccuFFicas software by trained personnel from the software manufacturer. Three personnel participated in the internal analysis and review process, and a single final FF value was generated for each patient according to the standardized workflow provided by the manufacturer. For ICAS, angio-based FF was defined as the pressure ratio distal to and proximal to the lesion. The following provides a summary of the core steps. (1) The image algorithm automatically selected the frame with the sharpest lumen contour following the importation of single angiographic view images. (2) Select the distal and proximal endpoints of the target vessel. The image algorithm automatically extracted the vessel centerline and lumen contour, with manual correction if necessary. (3) Based on the extracted vessel lumen region, patient-specific flow velocity of the target vessel was automatically acquired using the TIMI frame count method. (4) The flow velocity was employed as inlet boundary conditions, and a fast blood flow calculation algorithm was used to solve the fluid dynamic equations, thereby obtaining the pressure distribution of the target vessel and the FF value at the lesion. Blood was modeled as an incompressible Newtonian fluid with a density of 1,056 kg/m3 and viscosity of 0.0035 kg/(m·s). Simultaneously, the inlet pressure was the average arterial blood pressure. To ensure optimal FF measurement reproducibility, 2D DSA images were acquired at a frame rate of 12 frames per second (fps). This frame rate is necessary to achieve the most accurate TIMI frame count calculations, which directly influence the accuracy of the FF value. Variations in frame rates or angiographic projections could affect FF reproducibility, and these protocol-level considerations should be taken into account when applying AccuFFicas in clinical settings.

### Statistical analysis

Data analysis was performed using R software (version 4.4.2). First, normality tests (Shapiro–Wilk test) were conducted for continuous variables in the baseline characteristics. For normally distributed variables, independent-sample *t*-tests were used for intergroup comparisons. For non-normally distributed variables, the Wilcoxon rank-sum test was applied. Categorical variables were assessed for intergroup differences using the chi-square test or Fisher’s exact test (when expected frequencies were less than 5). Given the exploratory nature of this retrospective study, no formal adjustment for multiple comparisons was applied, and *p* values were interpreted as hypothesis-generating and were interpreted as hypothesis-generating. Additionally, multivariable analyses were performed to assess the association between FF, DS%, and clinical outcomes, adjusting for potential confounders including age, sex, diabetes, hypertension, hyperlipidemia, coronary artery disease, stroke history, smoking, alcohol consumption, and lesion location. The relationship between FF and DS% was further explored using scatter plot analysis and regression models. Patients with missing baseline data or those lost to follow-up were excluded to ensure data completeness. An exploratory logistic regression analysis including treatment modality, FF group, and their interaction term was performed to assess whether the association between treatment modality and stable or improved mRS outcomes at 12 months differed across FF strata. *Post hoc* sensitivity analyses compared mRS improvement, worsening, and functional independence within FF strata using two-sided Fisher’s exact tests. Because patients with discharge mRS 0 could not improve further, improvement was also assessed among those with discharge mRS ≥ 1. Absolute differences in proportions were reported with Newcombe 95% confidence intervals.

## Results

### Patient characteristics

A total of 257 patients were initially identified from the single-center database. Of these, 111 patients were excluded because of incomplete data or unmeasurable FF values (*n* = 75), or multiple intracranial arterial stenoses or concomitant intracranial aneurysms (*n* = 36). Among the remaining 146 eligible patients, 14 were lost to follow-up, leaving 132 patients for the final analysis (Figure 2).

**Figure 2:**
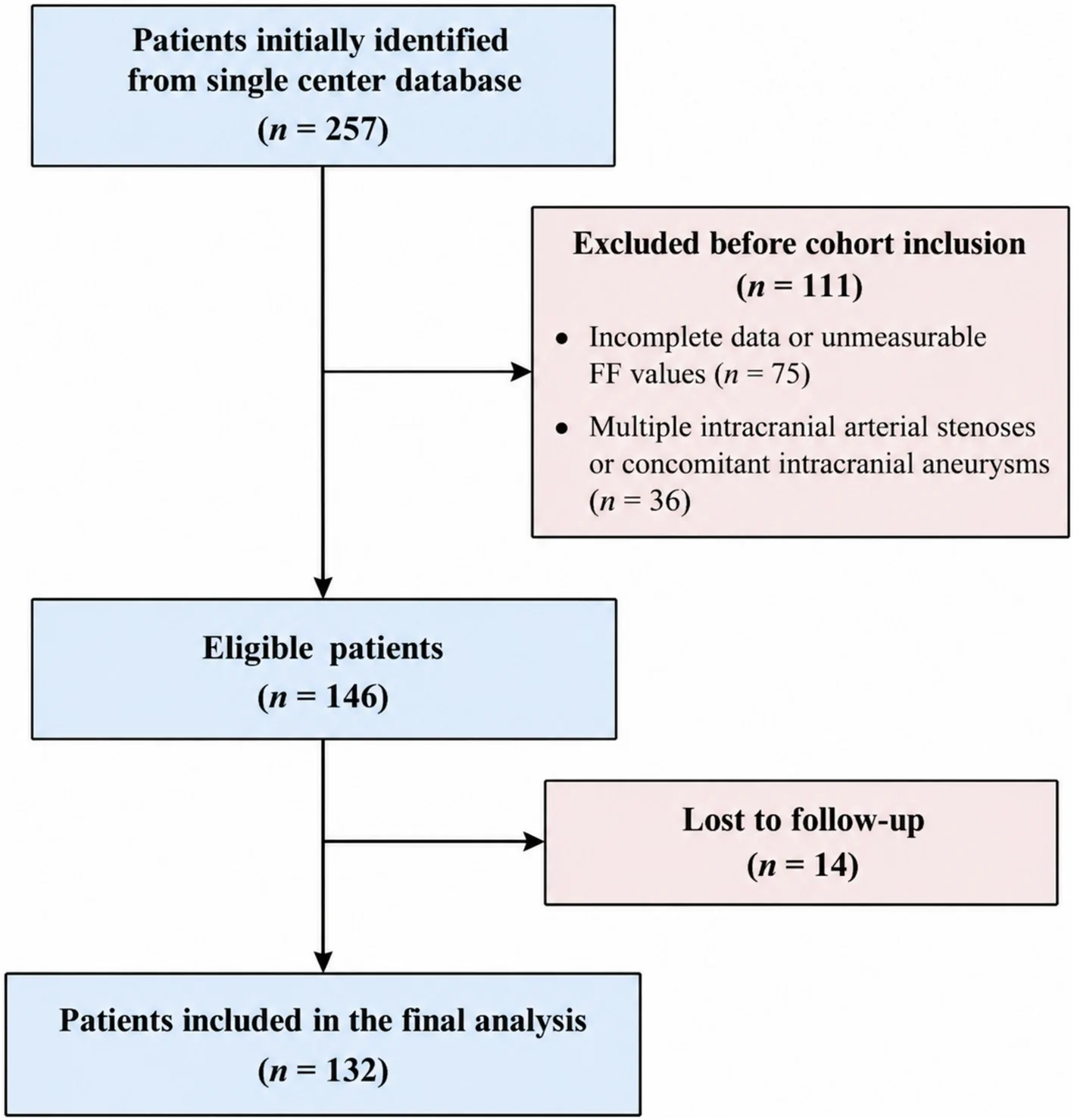
Flowchart of patient selection. The flowchart illustrates the screening, exclusion, follow-up, and final inclusion of patients in this retrospective study. FF, fractional flow.

Among the 76 patients who received endovascular treatment, 62 underwent intracranial stenting and 14 underwent balloon angioplasty alone. One patient had contrast extravasation; no serious periprocedural complications were recorded. The implanted stents comprised 39 Enterprise, 7 Enterprise 2, and 16 other stents, including Nova and Neuroform EZ. The final cohort was stratified into two groups based on pretreatment FF values: FF ≤ 0.76 (*n* = 82) and FF > 0.76 (*n* = 50). In the FF ≤ 0.76 group, 54 patients underwent interventional treatment and 28 received conservative management. In the FF > 0.76 group, 22 patients underwent interventional treatment and 28 received conservative management. There were no significant differences between treatment groups in terms of age, discharge mRS and NIHSS scores, or vascular risk factors, including diabetes, hypertension, smoking, and alcohol consumption (all *p* > 0.05) (Table 1). Similarly, in the subgroup of patients with DS% ≥ 70%, baseline characteristics were comparable between treatment groups (Supplementary Table 1). The distribution of discharge mRS scores differed between treatment groups within the FF ≤ 0.76 stratum: 10 of 54 patients (18.5%) in the interventional group and 17 of 28 (60.7%) in the conservative group had a discharge mRS score of 0.

**Table 1:** Patient characteristics and discharge status according to FF group.

Group	FF ≤ 0.76 *n* = 82	FF > 0.76 *n* = 50	*P*	FF ≤ 0.76 Interventional *n* = 54	FF ≤ 0.76 Conservative *n* = 28	*P*	FF > 0.76 Interventional *n* = 22	FF > 0.76 Conservative *n* = 28	*P*
Female	58 (70.7)	33 (66.0)	0.707	38 (70.4)	20 (71.4)	1.000	13 (59.1)	20 (71.4)	0.540
Age, year	60.95 ± 11.53	63.08 ± 9.98	0.281	61.65 ± 10.89	59.61 ± 12.77	0.451	63.00 ± 9.89	63.14 ± 10.23	0.961
Discharge mRS	1.29 ± 1.26	1.10 ± 1.42	0.418	1.48 ± 1.19	0.93 ± 1.33	0.059	1.50 ± 1.54	0.79 ± 1.26	0.077
Discharge NIHSS	1.68 ± 2.38	1.56 ± 2.46	0.777	1.80 ± 2.20	1.46 ± 2.73	0.553	2.23 ± 2.67	1.04 ± 2.19	0.089
Diabetes	33 (40.2)	17 (34.0)	0.594	22 (40.7)	11 (39.3)	1.000	10 (45.5)	7 (25.0)	0.224
Hypertension	53 (64.6)	34 (68.0)	0.836	37 (68.5)	16 (57.1)	0.436	16 (72.7)	18 (64.3)	0.742
Hyperlipidemia	5 (6.1)	3 (6.0)	1.000	4 (7.4)	1 (3.6)	0.840	3 (13.6)	0 (0.0)	0.157
Coronary heart disease	3 (3.7)	5 (10.0)	0.269	2 (3.7)	1 (3.6)	1.000	2 (9.1)	3 (10.7)	1.000
Identified stroke history	13 (15.9)	6 (12.0)	0.722	7 (13.0)	6 (21.4)	0.499	3 (13.6)	3 (10.7)	1.000
Smoking	24 (29.3)	12 (24.0)	0.647	19 (35.2)	5 (17.9)	0.168	6 (27.3)	6 (21.4)	0.883
Drinking	17 (20.7)	6 (12.0)	0.295	10 (18.5)	7 (25.0)	0.690	2 (9.1)	4 (14.3)	0.902
*Pre-FF*	0.49 ± 0.14	0.89 ± 0.06	/	0.47 ± 0.14	0.51 ± 0.15	0.334	0.89 ± 0.06	0.89 ± 0.07	0.962
Main symptoms									
Headache/Dizziness	24 (29.3)	20 (40.0)	0.281	14 (25.9)	10 (35.7)	0.504	8 (36.4)	12 (42.9)	0.861
Limb weakness	9 (11.0)	6 (12.0)	1.000	7 (13.0)	2 (7.1)	0.669	1 (4.5)	5 (17.9)	0.318
Numbness	44 (53.7)	22 (44.0)	0.370	26 (48.1)	18 (64.3)	0.248	13 (59.1)	9 (32.1)	0.106
Speech problems	22 (26.8)	14 (28.0)	1.000	17 (31.5)	5 (17.9)	0.290	6 (27.3)	8 (28.6)	1.000
Cognitive impairment	5 (6.1)	2 (4.0)	0.903	3 (5.6)	2 (7.1)	1.000	1 (4.5)	1 (3.6)	1.000
TIA	5 (6.1)	4 (8.0)	0.948	3 (5.6)	2 (7.1)	1.000	1 (4.5)	3 (10.7)	0.785
Atrial fibrillation	5 (6.1)	1 (2.0)	0.506	4 (7.4)	1 (3.6)	0.840	0 (0.0)	1 (3.6)	1.000
Lesion location			0.057			0.655			0.897
ICA	9 (11.0)	6 (12.0)		6 (11.1)	3 (10.7)		2 (9.1)	4 (14.3)	
MCA	62 (75.6)	28 (56.0)		39 (72.2)	23 (82.1)		13 (59.1)	15 (53.6)	
VA	4 (4.9)	8 (16.0)		3 (5.6)	1 (3.6)		4 (18.2)	4 (14.3)	
BA	7 (8.5)	8 (16.0)		6 (11.1)	1 (3.6)		3 (13.6)	5 (17.9)	

Discharge mRS and NIHSS were assessed after treatment; other clinical characteristics were recorded at admission unless otherwise specified.

Multivariable analyses were performed to assess the association between FF, DS%, and improvements in mRS and NIHSS scores within both the conservative and interventional treatment groups, adjusting for potential confounders including age, sex, diabetes, hypertension, hyperlipidemia, coronary artery disease, stroke history, smoking, alcohol consumption, and lesion location. No significant associations were observed between FF or DS% and improvements in mRS or NIHSS scores (all *p* > 0.1). Detailed results for the conservative and interventional treatment groups are shown in Supplementary Figures S1, S2, respectively.

### Outcomes stratified by FF

#### Pretreatment FF ≤ 0.76 group

Among patients with pretreatment FF ≤ 0.76, 54 underwent interventional treatment and 28 received conservative management.

Among patients with pretreatment FF ≤ 0.76, the proportion with no worsening in mRS between discharge and 12 months was 52 of 54 (96.3%) in the interventional group and 22 of 28 (78.6%) in the conservative group (Fisher’s exact *p* = 0.017; Figure 3a). This outcome combines patients whose scores improved with those whose scores remained unchanged. The separate analyses of improvement, unchanged scores, and worsening are reported below. No significant difference in NIHSS improvement was observed between the interventional and conservative groups (*p* = 0.331) (Figure 3b).

**Figure 3:**
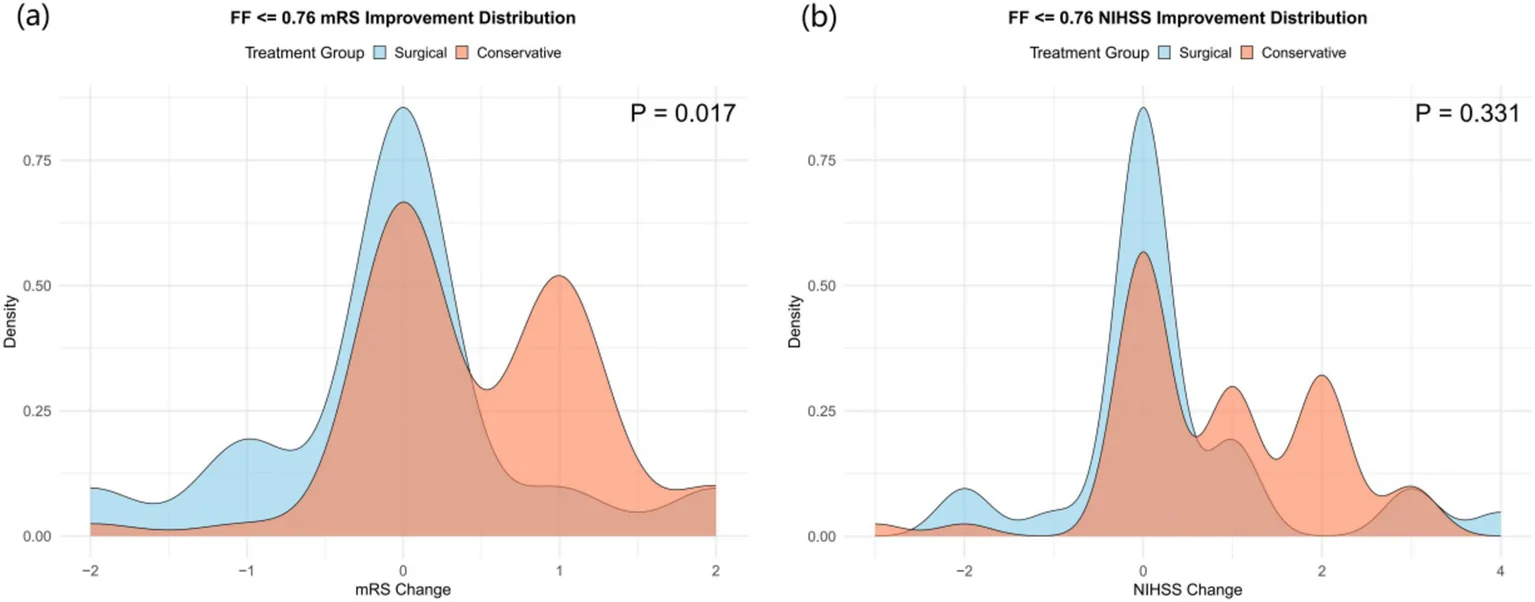
Clinical outcomes stratified by pretreatment FF (FF ≤ 0.76). **(a)** Distribution of mRS change in the interventional and conservative treatment groups. The proportion of patients with stable or improved mRS at 12 months was higher in the interventional group than in the conservative group (*p* = 0.017). **(b)** Distribution of NIHSS improvement, with no significant difference between treatment groups (*p* = 0.331).

#### Pretreatment FF > 0.76 group

Among patients with pretreatment FF > 0.76, 22 underwent interventional treatment and 28 received conservative management.

No significant difference in mRS improvement was observed between the interventional and conservative groups (*p* = 1.000) (Figure 4a). The distribution of mRS outcomes was similar between the two groups, with improvements concentrated within a relatively narrow range. Similarly, no significant difference in NIHSS improvement was observed between the two groups (*p* = 0.575) (Figure 4b). An exploratory interaction analysis did not identify a statistically significant treatment × FF group interaction for stable or improved mRS outcomes at 12 months (OR = 9.22, 95% CI: 0.66–129.69; *p* = 0.100).

**Figure 4:**
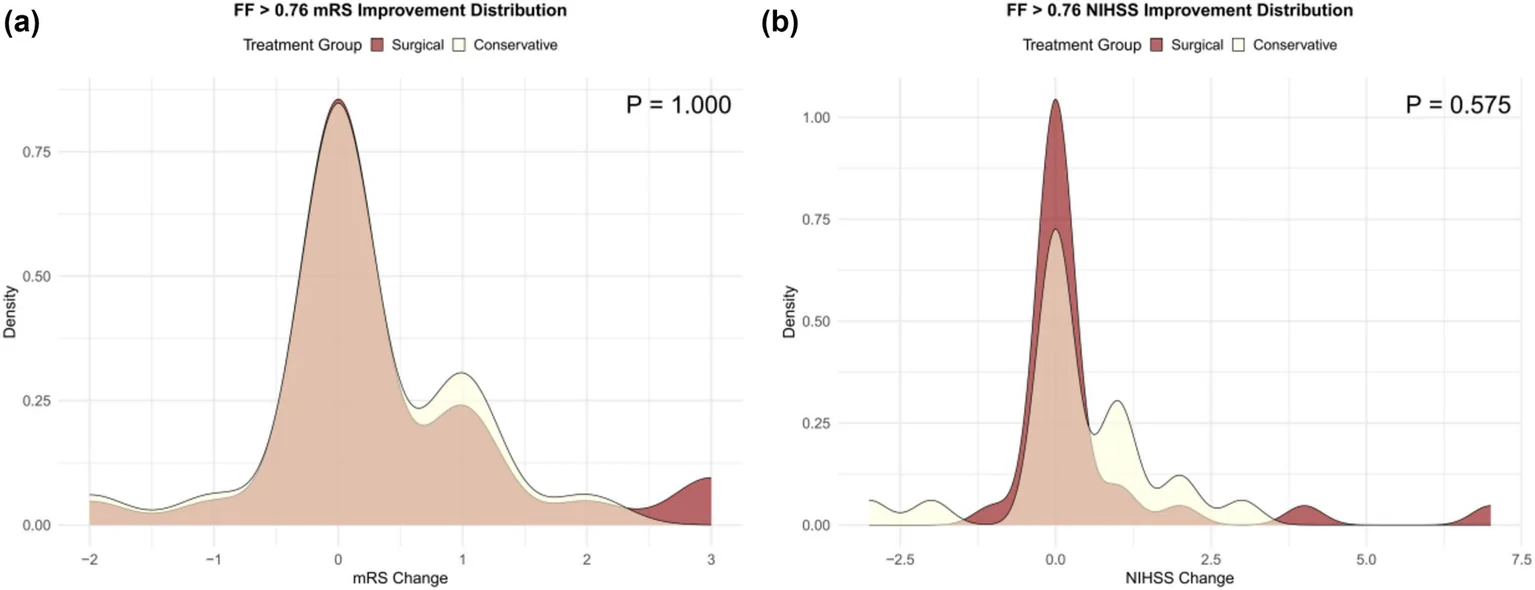
Clinical outcomes stratified by pretreatment FF (FF > 0.76). **(a)** mRS change distribution for FF > 0.76, with no statistically significant difference between the interventional and conservative treatment groups (*p* = 1.000). **(b)** NIHSS improvement distribution for FF > 0.76, with no statistically significant difference between the two groups (*p* = 0.575).

### Sensitivity analyses of functional outcomes

In the FF ≤ 0.76 stratum, mRS improved by at least one point between discharge and 12 months in 25 of 54 patients (46.3%) receiving interventional treatment and 5 of 28 patients (17.9%) receiving conservative treatment (Fisher’s exact *p* = 0.015; absolute difference, 28.4 percentage points; 95% CI, 6.7–44.9). Scores were unchanged in 27 and 17 patients, respectively, and worsened in 2 and 6 patients. Among patients with discharge mRS ≥ 1, improvement occurred in 25 of 44 (56.8%) and 5 of 11 (45.5%) patients, respectively (Fisher’s exact *p* = 0.521). Functional independence at 12 months (mRS 0–2) was observed in 46 of 54 (85.2%) and 24 of 28 (85.7%) patients, respectively. In the FF > 0.76 stratum, improvement of at least one point occurred in 6 of 22 (27.3%) and 8 of 28 (28.6%) patients, respectively (Fisher’s exact *p* = 1.000). The distributions of mRS scores at discharge and 12 months are shown in Supplementary Figure S3.

### Analyses based on DS%

#### Exploration of FF and DS% relationship

The relationship between FF and DS% was evaluated using scatter plot analysis and regression modeling (Figure 5). Although a negative trend was observed, indicating that FF tended to decrease as DS% increased, the association was not statistically significant (*p* = 0.221). The low R-squared value (*R*^2^ = 0.011) suggests that DS% explains only a small proportion of the variability in FF. The data points were widely dispersed, with no clear linear or non-linear pattern observed.

**Figure 5:**
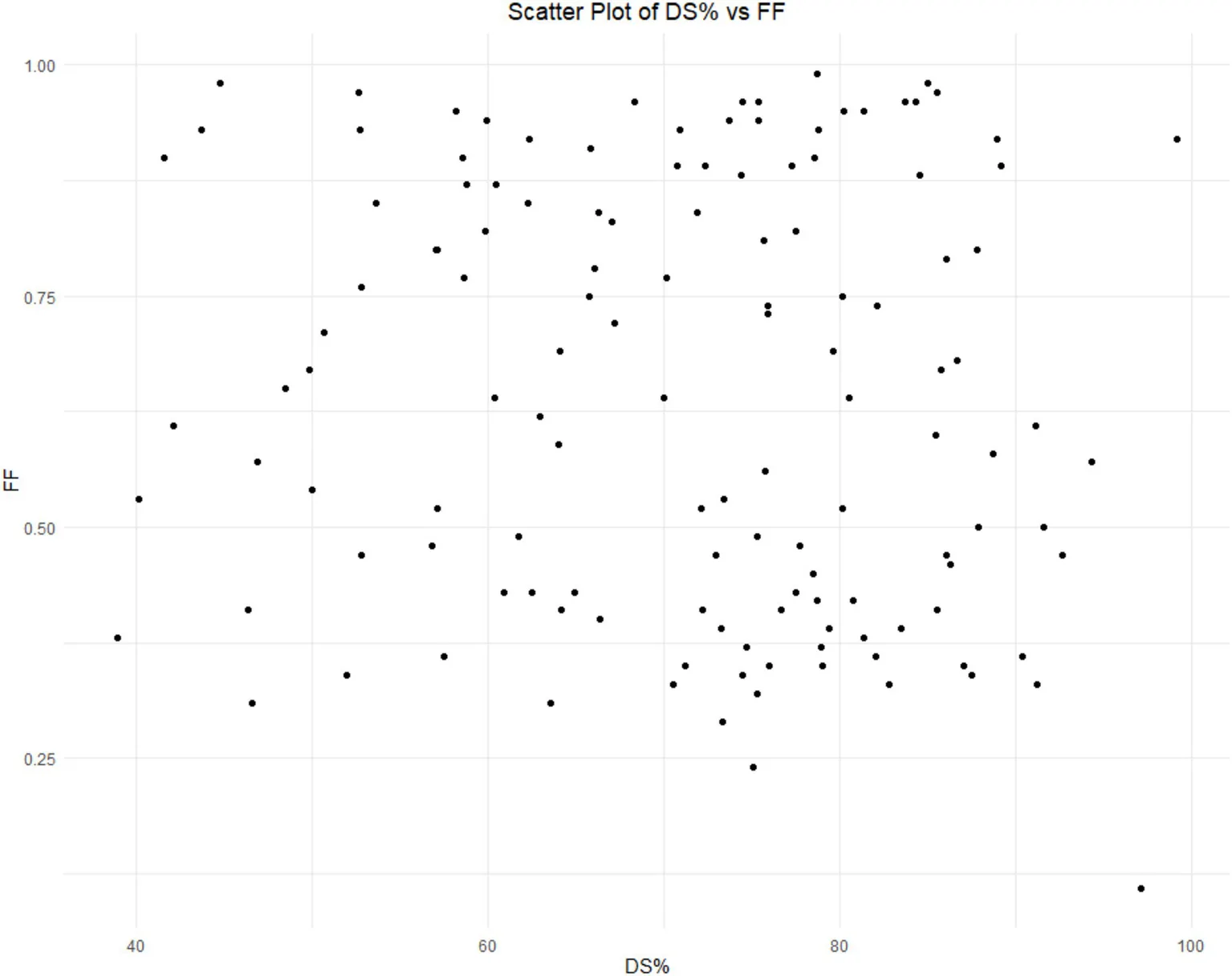
Scatter plot of DS% versus FF. Scatter plot showing the relationship between DS% (degree of stenosis) and FF (fractional flow) values. A linear regression model was applied to explore the relationship between DS% and FF. Although the regression model suggested a negative association (FF decreases as DS% increases), the *p*-value for the relationship (*p* = 0.221) indicates that this association is not statistically significant. The low R-squared value (0.01149) suggests that DS% explains only a small portion of the variation in FF.

#### Example of discordance between FF and DS%

A representative case of discordance between DS% and FF is shown in Figure 6. The degree of stenosis was 74.37%, indicating severe anatomical narrowing, while the corresponding FF value was 0.79. The patient also exhibited vessel tortuosity, which may affect morphological assessment. Despite the high degree of stenosis, the FF value remained relatively preserved, reflecting a discrepancy between anatomical severity and functional assessment.

**Figure 6:**
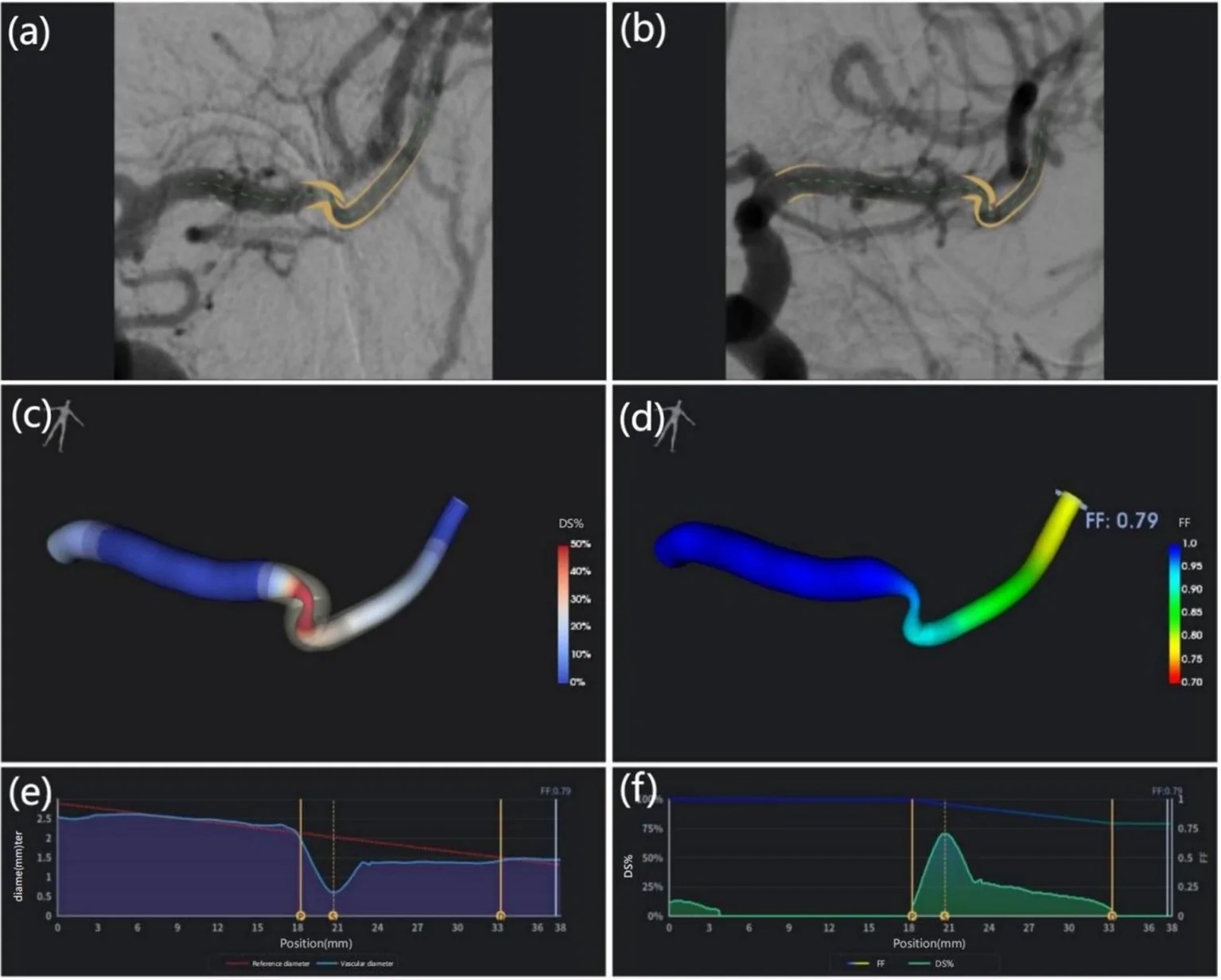
Example of discordance between FF and DS% in symptomatic ICAS. The upper panels show the angiographic images **(a,b)** and 3D reconstruction of the arterial segment **(c)**, with DS% values visualized in a color gradient. The other panels display the vascular assessment: **(d)** FF calculation at the lesion site, with FF = 0.79. **(e)** Changes in vascular diameter along the vessel, showing the reference diameter and actual vascular diameter. **(f)** Changes in DS% (degree of stenosis) along the vessel, highlighting the stenosis severity at different points.

#### Preoperative DS% ≥ 70% group

In patients with DS% ≥ 70%, no significant differences were observed in the distributions of mRS improvement between the interventional and conservative treatment groups (*p* = 0.688) (Figure 7a). Similarly, no significant difference in NIHSS improvement was observed between the two groups (*p* = 0.310) (Figure 7b).

**Figure 7:**
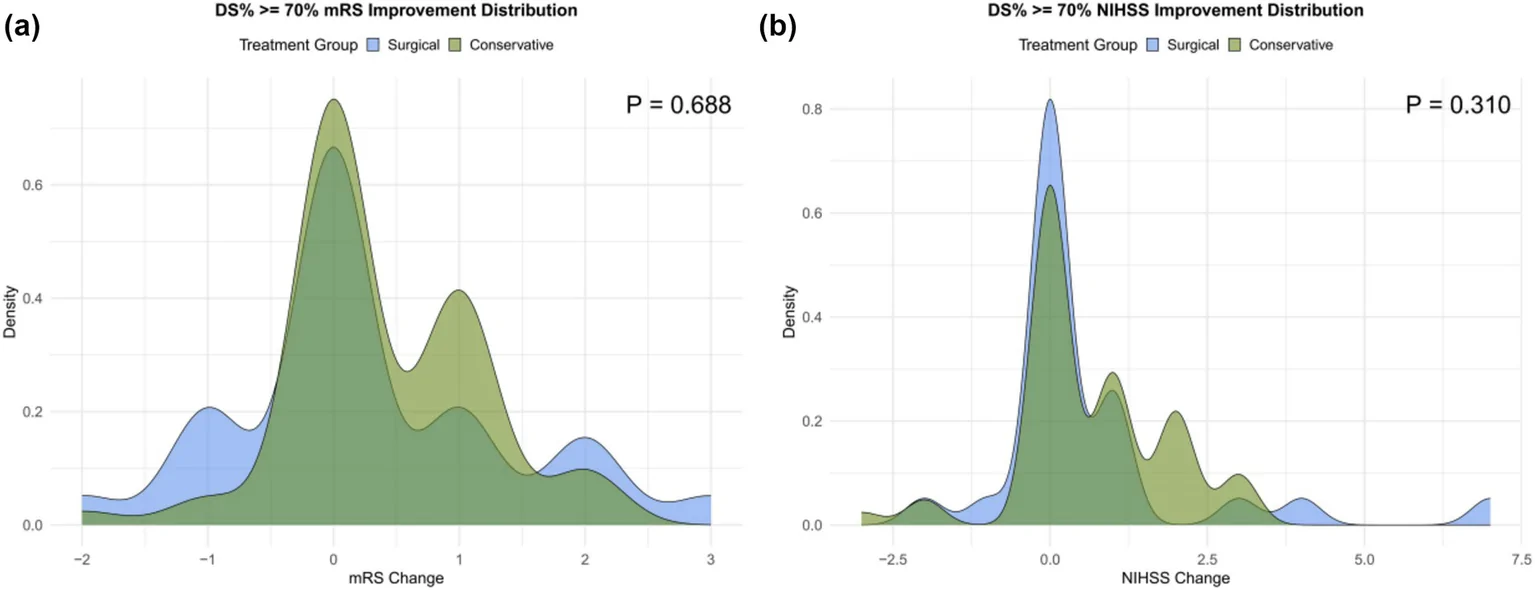
Clinical outcomes stratified by DS% (DS% ≥ 70%). **(a)** mRS improvement distribution for DS% ≥ 70%, showing no statistically significant difference between the interventional and conservative treatment groups (*p* = 0.688). **(b)** NIHSS improvement distribution for DS% ≥ 70%, with no statistically significant difference between the two groups (*p* = 0.310).

Although the interventional treatment group showed a relatively higher concentration of mRS improvement than the conservative group, this difference did not reach statistical significance. In this cohort, DS%-based stratification did not identify statistically significant differences in clinical outcomes between the treatment groups.

## Discussion

In this exploratory cohort, patients with FF ≤ 0.76 who received interventional treatment more often had no mRS worsening from discharge to 12 months than those receiving conservative treatment. In a *post hoc* analysis of patients with FF ≤ 0.76, mRS improved by at least one point in 46.3% of the interventional group and 17.9% of the conservative group. However, discharge functional status differed substantially between groups: more conservatively treated patients had mRS 0 and thus could not improve further. The difference in improvement was not statistically significant among patients with discharge mRS ≥ 1, although this comparison involved few patients. Neither the adjusted analyses nor the treatment-by-FF interaction provided convincing evidence that FF identifies patients who benefit more from intervention. Treatment selection was nonrandomized, and these findings should be interpreted as exploratory associations.

Currently, DS% is widely used in clinical practice to guide the diagnosis and treatment of ICAS patients. However, it has several limitations. In cases of severe ICAS, only a proportion of patients exhibit hemodynamic significance, suggesting that DS% alone may not fully capture the clinical implications of stenosis (20). Additionally, DS% is based on static anatomical assessment and does not reflect dynamic changes in cerebral blood flow or the patient’s compensatory capacity. As a result, reliance on anatomical severity alone may be insufficient to fully characterize disease impact in ICAS (21).

In this context, functional assessment has gained increasing attention, as it may provide complementary information regarding the physiological significance of vascular lesions. In the present study, we focused on symptomatic ICAS patients and incorporated FF as a functional parameter to evaluate hemodynamic status. Our findings suggest that FF-based stratification may provide complementary information beyond DS% for exploring associations between treatment strategies and clinical outcomes. Importantly, DS% remains a cornerstone in clinical decision-making, particularly when interpreted in conjunction with clinical symptoms and other imaging findings. Large clinical trials such as SAMMPRIS and VISSIT have demonstrated its role in identifying patients at higher risk and guiding treatment strategies. Therefore, FF should not be considered a replacement for DS%, but rather a complementary measure that may improve patient stratification when integrated with existing clinical assessments.

In recent years, increasing attention has been paid to the assessment of cerebral hemodynamics in cerebrovascular diseases. Techniques such as perfusion-weighted magnetic resonance imaging (PWI) and transcranial Doppler (TCD) are widely used to evaluate cerebral blood flow and perfusion status. However, these methods may be influenced by factors such as bilateral lesions, posterior circulation involvement, and heterogeneous flow distribution (22). In this context, functional indices derived from angiographic imaging, such as FF, have been proposed to provide additional information on the physiological impact of vascular stenosis. FF reflects the pressure gradient across a lesion and may offer insight into the hemodynamic significance of intracranial arterial narrowing (17, 23). Previous studies have suggested that reduced FF values may be associated with impaired cerebral perfusion reserve and an increased risk of ischemic events (24, 25). In line with these observations, our findings indicate that FF-based stratification was associated with differences in functional outcomes across treatment strategies.

At present, there is no standardized FF threshold for guiding clinical decision-making in ICAS. In the present study, we explored the potential relevance of an FF cutoff value in relation to treatment response. In the field of coronary artery disease, FFR thresholds such as 0.75 and 0.80 have been established through extensive validation studies (26–28). By contrast, intracranial FF research remains limited, and previously reported thresholds have varied considerably. For example, Wang et al. reported that FF ≤ 0.67 was associated with restricted blood flow, while Ge et al. suggested that FF ≤ 0.9 may indicate insufficient downstream perfusion (17, 29). However, these studies were based on small sample sizes and primarily focused on hemodynamic measurements rather than long-term clinical outcomes. In our previous work, an FF value of 0.76 demonstrated the highest agreement between AccuFFicas-derived measurements and guidewire-based assessments (18). Based on this observation, we adopted 0.76 as a reference value for stratification in the present study. Our findings provide preliminary clinical observations suggesting that this threshold may be associated with differences in functional outcomes across treatment strategies. However, this cutoff was not derived from outcome-based optimization and should therefore be interpreted with caution. Further studies with larger sample sizes, multicenter designs, and longer follow-up are required to validate the clinical applicability of this threshold. In addition, integrating FF with other hemodynamic parameters and imaging biomarkers may further improve its role in clinical assessment.

This study has several limitations. First, this was a single-center retrospective study with a relatively small sample size, which may limit the generalizability of the findings. Larger, multicenter prospective studies are needed to further validate the role of FF in ICAS. Second, the primary comparison used a composite outcome of no worsening, which included both improvement and unchanged scores. A statistically significant difference in this composite outcome does not establish a clinically meaningful improvement in mRS. We therefore added *post hoc* analyses that separated improvement, unchanged scores, and worsening; these analyses remain limited by unequal discharge functional status and the small subgroup sizes. Third, the FF threshold of 0.76 used in this study was not derived from outcome-based optimization, and its clinical applicability therefore requires further validation. Finally, although patients were followed for 12 months, longer-term outcome data were not available and should be explored in future studies. In addition, because treatment allocation was based on clinical judgment rather than randomization, residual and unmeasured confounding cannot be excluded. Collateral status was not systematically graded, and perfusion imaging was not available for all patients; these factors could not be consistently included in the adjusted analyses. Therefore, the observed differences between treatment strategies should be interpreted as associations rather than causal treatment effects. In addition, individual operator-level FF measurements were not available, precluding a formal assessment of inter-operator agreement in this retrospective study. Future prospective studies should evaluate FF measurement reproducibility across independent operators.

In conclusion, this exploratory study suggests that FF-based stratification may provide additional information beyond conventional anatomical assessment in patients with symptomatic ICAS. Patients with FF ≤ 0.76 appeared to show different functional outcomes across treatment strategies, whereas DS%-based stratification showed limited discriminatory value in this cohort. However, these findings should be interpreted cautiously given the retrospective single-center design, limited sample size, and the exploratory nature of the FF threshold. Larger prospective studies are needed to validate the clinical utility of FF in treatment stratification.

## Data Availability

The raw data supporting the conclusions of this article will be made available by the authors, without undue reservation.
