# Supplementary material for: Angiography-derived fractional flow and clinical outcomes in symptomatic intracranial atherosclerotic stenosis
Source: Front Neurol. 2026 Oct 15;17:1944177. doi: 10.3389/fneur.2026.1944177 (PMC13716638; doi:10.3389/fneur.2026.1944177)
Supplement: Supplementary file 1 [file Supplementary_file_1.doc]

**Supplementary Figure S1. Associations of fractional flow and degree of stenosis with changes in neurological outcomes in the conservative treatment group.**

(a) Fractional flow (FF) and change in modified Rankin Scale (mRS) score; (b) degree of stenosis (DS%) and change in mRS score; (c) FF and change in National Institutes of Health Stroke Scale (NIHSS) score; (d) DS% and change in NIHSS score. Points represent individual patients. Solid lines show unadjusted linear fits, with shaded 95% confidence intervals; the P values shown in each panel are from the adjusted models. Negative changes indicate improvement.

**Supplementary Figure S2. Associations of fractional flow and degree of stenosis with changes in neurological outcomes in the interventional treatment group.**

(a) FF and change in mRS score; (b) DS% and change in mRS score; (c) FF and change in NIHSS score; (d) DS% and change in NIHSS score. Points represent individual patients. Solid lines show unadjusted linear fits, with shaded 95% confidence intervals; the P values shown in each panel are from the adjusted models. Negative changes indicate improvement.

**Supplementary Figure S3.** **Distribution of modified Rankin Scale scores at discharge and 12 months by FF stratum and treatment group.**

Stacked bars show the percentage of patients at each mRS score; numbers within segments indicate patient counts. No patients had an mRS score of 5 or 6 at either assessment.
