# Supplementary material for: Angiography-derived fractional flow and clinical outcomes in symptomatic intracranial atherosclerotic stenosis
Source: Front Neurol. 2026 Oct 15;17:1944177. doi: 10.3389/fneur.2026.1944177 (PMC13716638; doi:10.3389/fneur.2026.1944177)
Supplement: Supplementary file 2 [file Table_1.doc]

**Supplementary Table.1 Baseline characteristics for DS%≥**70% group

| Group | High Stenosis Interventional Group n=55 | High Stenosis Conservative Group n=26 | P |
| --- | --- | --- | --- |
| Female | 39 (70.9) | 19 (73.1) | 1.000 |
| Age, year | 62.24 (10.28) | 61.54 (11.32) | 0.783 |
| Discharge mRS | 1.38 (1.30) | 1.00 (1.26) | 0.216 |
| Discharge NIHSS | 1.78 (2.34) | 1.19 (2.12) | 0.279 |
| diabetes | 24 (43.6) | 10 (38.5) | 0.842 |
| hypertension | 39 (70.9) | 18 (69.2) | 1.000 |
| Hyperlipidemia | 5 (9.1) | 1 (3.8) | 0.699 |
| Coronary heart disease | 4 (7.3) | 3 (11.5) | 0.830 |
| Identified Stroke History | 7 (12.7) | 5 (19.2) | 0.664 |
| Smoking | 21 (38.2) | 6 (23.1) | 0.274 |
| drinking | 11 (20.0) | 5 (19.2) | 1.000 |
| Pre-FF | 0.60 (0.24) | 0.65 (0.25) | 0.379 |
| **Main symptoms** | | | |
| Headache/Dizziness | 16 (29.1) | 11 (42.3) | 0.355 |
| Limb weakness | 7 (12.7) | 4 (15.4) | 1.000 |
| Numbness | 25 (45.5) | 14 (53.8) | 0.640 |
| Speech problems | 18 (32.7) | 8 (30.8) | 1.000 |
| Cognitive impairment | 2 (3.6) | 2 (7.7) | 0.812 |
| TIA | 3 (5.5) | 2 (7.7) | 1.000 |
| Atrial fibrillation | 4 (7.3) | 2 (7.7) | 1.000 |
| Lesion location |  |  | 0.808 |
| ICA | 5 (9.1) | 1 (3.8) |  |
| MCA | 37 (67.3) | 18 (69.2) |  |
| VA | 6 (10.9) | 4 (15.4) |  |
| BA | 7 (12.7) | 3 (11.5) |  |
